# Intra-familial transmission of Hepatitis B virus in a peri-urban community from the Democratic Republic of the Congo

**DOI:** 10.1186/s41182-025-00781-x

**Published:** 2025-07-28

**Authors:** Florence Cindibu Kalonji, Yu Nakagama, Evariste Tshibangu-Kabamba, Nadine Kayiba Kalenda, Shun Nakagama, Sachie Nakagama, Pathy Kamanga Nkolongo, Nestor Kalala-Tshituka, Alphonse Lufuluabu Mpemba, Faustin Ndjibu Mpoji, André Kabongu Kalala, Benjamin Muamba Mpoyi, Dieudonné Mumba Ngoyi, Natsuko Kaku, Yusuke Shimakawa, Ghislain Tumba Disashi, Yasutoshi Kido

**Affiliations:** 1 Department of Virology and Parasitology, Graduate School of Medicine, Osaka Metropolitan University, 1-4-3 Asahi-Cho, Abeno-Ku, Osaka-Shi, Osaka 545-8585 Japan; 2 Department of Internal Medicine, Faculty of Medicine-Pharmacy and Public Health, University of Mbujimayi, Mbujimayi, Democratic Republic of Congo; 3 Department of Public Health, Faculty of Medicine-Pharmacy and Public Health, University of Mbujimayi, Mbujimayi, Democratic Republic of Congo; 4 Secondary Hospital of Katanda, Mbujimayi, Kasaï-Oriental Democratic Republic of Congo; 5 Department of Parasitology, National Institute of Biomedical Research, Kinshasa, Democratic Republic of Congo; 6 Institut Pasteur, Université Paris Cité, Unité d’Épidémiologie des Maladies Émergentes, Paris, France

**Keywords:** Intra-familial, Transmission, Hepatitis B virus, Peri-urban, Community, Democratic Republic of Congo

## Abstract

**Background:**

Despite global elimination targets set for 2030, Hepatitis B virus (HBV) infection remains a major public health challenge in low-income countries, including the Democratic Republic of Congo (DRC). Limited evidence on the regional transmission pathways precludes progress towards HBV elimination. This study aimed to assess the prevalence, molecular characteristics, and transmission dynamics of HBV in the Lukelenge health district, a peri-urban area in central DRC.

**Methods:**

We employed a two-tiered recruitment strategy: community member volunteers were enrolled during the first phase, and upon notification of HBV positivity in an index case, family contacts were subsequently recruited in the second phase. Participants were screened for hepatitis B surface antigen (HBsAg), followed by PCR amplification of HBV DNA and sequencing. Genotyping and phylogenetic analysis of preS/S sequences were performed to explore regional HBV diversity and transmission patterns.

**Results:**

A total of 751 participants from 677 households were included. The overall HBsAg prevalence was 3.8% [95% CI 2.6–5.7], with the highest rate (10.1% [95% CI 4.9–18.2]) found in children aged 5 years and younger. All 42 HBV isolates belonged to genotype E, with 97.6% sharing the *ayw4* serotype. Mutations with relevancy to immune escape were detected in 9.5% of strains, while those possibly linked to antiviral resistance were found in 4.7%. Maximum likelihood phylogenetic analysis showed intra-familial clustering of preS/S sequences, suggesting that parent-to-child transmission was the most frequent mode of HBV spread in the study population.

**Conclusions:**

HBV in Lukelenge shows intermediate endemicity, especially affecting young children. Intra-familial transmission is revealed to be predominant, likely involving both vertical and horizontal pathways. Family-targeted interventions, including maternal screening and universal birth-dose vaccination, should be prioritized to eliminate HBV in this region.

**Supplementary Information:**

The online version contains supplementary material available at 10.1186/s41182-025-00781-x.

## Background

Hepatitis B virus (HBV) infection remains a major worldwide health challenge, with an estimated 254 million chronically infected people, causing substantial mortality from liver cirrhosis and hepatocellular carcinoma (HCC) [1]. In response, the World Health Organization (WHO) set a goal to eliminate HBV infection by 2030, with a 90% reduction in new infections and a 65% reduction in deaths through expanded vaccination, early diagnosis, enhanced antiviral access, and the development of emerging curative therapies [2, 3]. In spite of these worldwide efforts, HBV infection persists, particularly in sub-Saharan Africa, which accounts for 63% of global new infections annually [3]. Africa faces substantial gaps in vaccination, diagnostics, and treatment, exacerbating the global burden of HBV, with significant disparities observed both across and within countries in the region [4, 5]. In the Democratic Republic of the Congo (DRC), HBV prevalence is in line with broader African trends. The national prevalence is reported to be around 3.3%, with approximately 300.000 children living with chronic hepatitis B, far exceeding the WHO’s 2030 elimination target of ≤ 0.1% prevalence in children under five [6–8]. Rural and peri-urban areas face the most notable gaps in HBV control strategies, necessitating urgent region-specific interventions [5, 9, 10].

Since HBV can be transmitted through blood, semen, or vaginal fluids via either horizontal or vertical routes, and the relative importance of each pathway varies geographically [11], it is essential to understand regional transmission dynamics in order to implement context-specific interventions. Horizontal transmission has been widely recognized in Africa, especially through close contact within households and communities [12]. However, with increased coverage of the three-dose hepatitis B vaccination series (HepB3) in infants, the significance of horizontal transmission would be diminishing [13]. In contrast, the relative importance of vertical transmission remains debated, especially in the DRC, where a study in pregnant women and their offspring has suggested a crucial role of mother-to-child transmission in the persistence and spread of HBV within households [14]. Population-based studies providing insight into this hypothesis remain limited in DRC.

Genetic analysis of HBV gives valuable insights into viral strain typing and transmission dynamics. The HBV genome is a circular, partially double-stranded DNA (~ 3.2 kb) comprising four overlapping open reading frames (ORFs): preS/S, preCore/Core, Pol, and X [15]. The preS/S ORF, which encodes for the surface antigen of hepatitis B (HBsAg), is highly variable and has been thoroughly studied for phylogenetic purposes [15, 16]. This region’s variability is partly driven by the overlapping Pol ORF, encoding a polymerase with low replication fidelity, yielding a high mutation rate. As a result, ten HBV genotypes (A–J), nine serotypes, and over 40 sub-genotypes have been identified [16, 17]. Mutations in the preS/S region, particularly within the “a” determinant of the Small S protein, are of critical clinical importance as they can give rise to immune escaping strains capable of infecting vaccinated individuals and causing occult HBV infections [15]. These mutations can also be accountable for antiviral drug resistance and disease progression to cirrhosis or HCC [18]. Overall, genetic analysis of the preS/S region is useful for understanding HBV diversity, transmission dynamics, and its implications on disease progression and treatment resistance [15, 16].

Comprehensive genetic analyses of HBV strains circulating among the African population are crucial for guiding the implementation of targeted public health interventions. However, such studies detailing HBV genotypes, mutations, and transmission pathways remain scarce in the DRC [19, 20]. To address this critical knowledge gap, the present study aimed to assess HBV prevalence, genetic diversity, and potential transmission patterns within a peri-urban community in central DRC.

## Methods

### Design and setting of the study

**Fig. 1:**
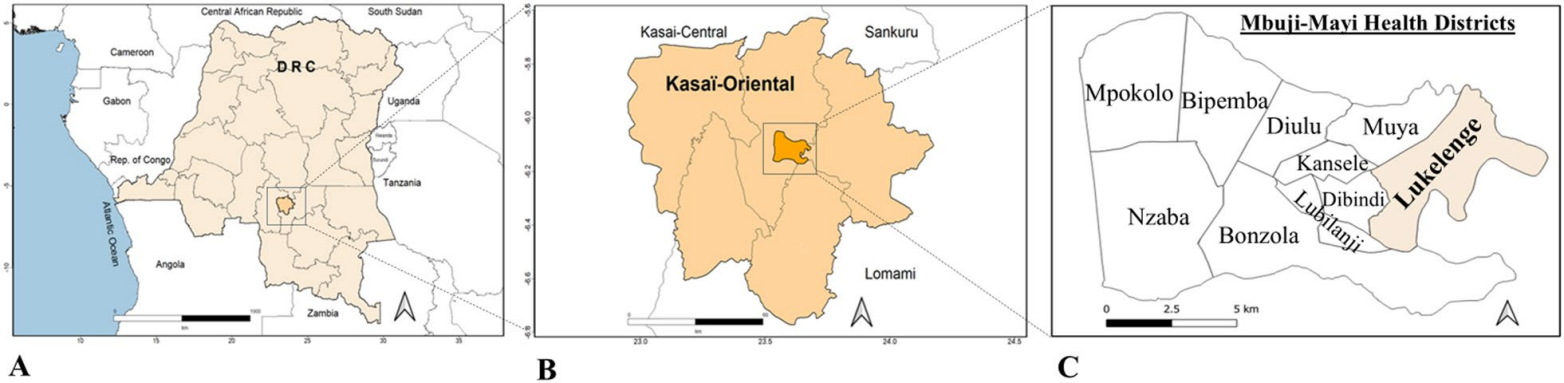
Geographic location of the study sites. Location of the Lukelenge health district at different geographical levels: the Democratic Republic of the Congo (**A**), the Kasaï-Oriental province (**B**), and the city of Mbujimayi (**C**)

This community-based cross-sectional study was conducted between April and November 2023 in the Lukelenge health district (HD), located in Mbujimayi, Kasaï-Oriental province, DRC, an area endemic for HBV (Fig. 1). Lukelenge, a peri-urban area on the outskirts of Mbujimayi, spans 35 km2 and has an estimated population of 341,591, resulting in a population density of approximately 9760 inhabitants per km^2^. The local economy is primarily based on artisanal diamond mining; however, declining profitability has considerably impacted household income, making Lukelenge one of the most impoverished HDs in the region [21].

### Sampling strategy and study population

“A simple random sampling method was used to select households in the study area, based on the 70.772 household’s registry provided by the Central Office of Lukelenge HD. A unique identifier was first assigned to each household, then a numerical sequence ranging from 1 to 70.772 was generated, constituting the sampling frame, using R software v4.4.3 (The R Development Core Team, Austria) [32]. The *sample()* function was used to draw a random sample of unique numbers, corresponding to the target sample size.”

Participant recruitment followed a two-tiered approach, with visits spaced 6 months apart. During the first visit, all selected households were visited, and within each household, one eligible individual, aged 1 year or older and residing in the study area for at least 3 months, was randomly selected using a lottery method to be screened for HBsAg. Those who tested positive for HBsAg were designated as “index cases” and their households were subsequently invited to participate in the second-phase, family-based screening. During the second visit, up to five household members, including the index case’s parents and siblings, were selected as “contact cases” for HBsAg screening. Index cases underwent HBsAg retesting to confirm chronic HBV infection prior to the inclusion of household members. Eligibility required written consent from adults, or permission from a parent or legal guardian for minors (under 18 years). Additionally, verbal assent was obtained from minors aged 15 to 18 years. Using Cochran’s formula [22], the required sample size for the community survey was calculated assuming a 3.3% estimated prevalence of HBsAg [6], a 95% confidence level (*Z* = 1.96), and an absolute margin of error of 1.4%. This yielded a minimum required sample size of 626 participants. To account for possible non-response, a final sample size of 700 was targeted.

### Field investigation and sample collection

Each visit involved a brief interview to assess eligibility, gather demographic data, and document the family pedigree. Eligible participants underwent a rapid HBsAg test (One Step HBsAg Rapid Test Strip, RapidLab, China) following the manufacturer's instructions. Briefly, 50 µL of blood was collected via fingerprick, applied to the test strip, and allowed to develop for 15 min. For HBV DNA extraction, an additional 50 µL of blood was collected, spotted onto a Whatman 903 filter card (Cytiva, USA), and allowed to dry. The dried blood spot sample (DBSs) was stored in a zip-lock bag with desiccants at ambient temperature. The samples were later transferred to the Biobank of the *Institut National de Recherche Biomédicale* (INRB) in Kinshasa, DRC, and stored at -20 °C. These DBSs were then used for molecular analyses in Japan. Specimens were not re-collected from the index case during the second visit. Field data were recorded using a semi-structured electronic questionnaire, designed with the KoboToolBox application (https://kf.kobotoolbox.org/). Participants who tested positive for HBsAg received counseling and were referred to the Lukelenge HD’s reference hospital for appropriate care and follow-up.

### HBV DNA extraction, PCR amplification, and sequencing

Total DNA was extracted from dried blood spot samples using the DNeasy® Blood and Tissue Kit (Qiagen, Hilden, Germany). The DNA was eluted in 30 µL of final volume. PCR reactions included 2 µL of DNA solution as template in a 25 µL total volume, and PrimeSTAR Max DNA Polymerase (TaKaRa, Japan), and were run on a C1000 Touch thermal cycler (Bio-Rad, USA). Previously established sets of primers were used (Supplementary Table S1), with PCR conditions that we optimized for HBV genomic DNA amplification, as indicated below:

1. PreS/S region amplification (~ 1200 bp): a semi-nested PCR was performed using primers PS1 and P3 for the first round, followed by PS1 and S2 for the second round [16], with a final primer concentration of 0.2 µM. Thermal cycling conditions consisted of 30 cycles at 98 °C for 10 s, 55 °C for 15 s, and 72 °C for 5 s.
2. Whole-genome amplification (~ 3200 bp): universal primers WA-L and WA-R [23], with a final concentration of 0.5 µM, were used. Thermal cycling conditions included 40 cycles at 98 °C for 10 s, 60 °C for 5 s, and 72 °C for 30 s. This was performed only on the index cases specimens.

Successfully amplified PCR products were purified using the NucleoFast® 96 PCR Clean-up kit (Macherey–Nagel, France) and subsequently sequenced using the 3730XL DNA Analyzer (Applied Biosystems) with specific sequencing primers detailed in Supplementary Table S1.

### HBV molecular analysis

Nucleotide sequences were inspected and trimmed using Chromas Software v2.6.6 [24]. They were assembled on the CLC Genomics Workbench platform v.23 (CLC bio, Denmark), and then aligned using MAFFT v7 [25]. Phylogenetic analysis was performed using maximum likelihood trees reconstructed with IQ-TREE v2.3.6 [26] and visualized as midpoint-rooted trees on the Interactive Tree of Life (iTOL) platform v7 [27]. Node support was evaluated using 1000 bootstrap replicates to assess topology robustness. The most appropriate nucleotide substitution model was selected based on the Bayesian Information Criterion (BIC). Reference sequences of HBV genotypes A to J were retrieved from the Genbank database (https://www.ncbi.nlm.nih.gov/genbank/) and incorporated into the phylogenetic trees. Genotyping was cross-validated using the Hepatitis B Virus Phylogenetic Typing Tool v2.59 [28]. Genetic distances within and between families were calculated using the Kimura-2-Parameter (K2P) substitution model in MEGA v11 [29]. HBV serotypes were inferred by analyzing key amino acid residues within the small S protein sequence (i.e., positions 122, 127, 140, 159, and 160), following established classification algorithms [30]. Mutations linked to immune escape or antiviral resistance were further predicted using the HIV-Grade: HBV-Tool [31], assessing clinically relevant viral polymorphisms.

### Statistical analysis

Statistical analyses were performed using R software version 4.4.3 (The R Development Core Team, Austria) [32]. Categorical variables were summarized as absolute and relative frequencies, while continuous variables were described using medians and ranges (minimum to maximum). The prevalence of HBsAg was estimated at both community and intra-familial levels using Jeffreys’ approximate Bayesian confidence intervals. Age-specific prevalences were calculated based on data from index case screening, without considering intra-familial screening data. The Chi-square test was used to compare HBsAg positivity rates between groups, and the odds ratio indicating the trend in the likelihood of positivity. Genetic distances of HBV within and between families were compared using the Wilcoxon rank-sum test. Receiver operating characteristic (ROC) curve analysis was applied to predict monophyletic clusters based on sequence similarity, in order to determine the optimal cutoff value for viral genetic divergence as the threshold for defining similarity between HBV sequences. A *p*-value of less than 0.05 was considered statistically significant.

## Results

### Community survey (among index cases)

**Fig. 2:**
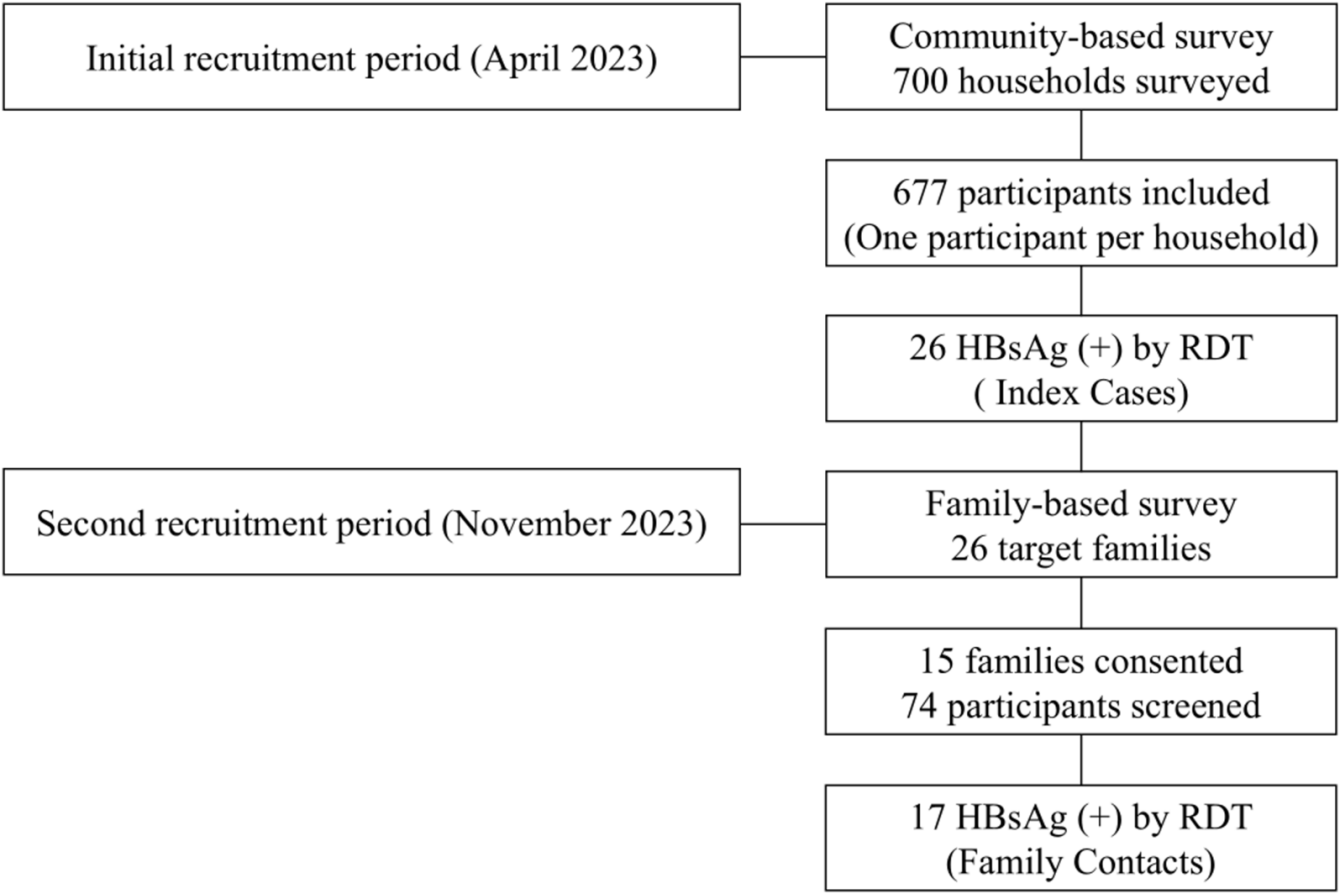
Participant recruitment and selection process in this study. *RDT* rapid diagnostic test, *HBsAg* Hepatitis B surface antigen, *(+)* positive

In the community survey, a total of 700 households were randomly selected, and 700 eligible participants were recruited, with 677 providing complete survey responses, yielding a response rate of 97% (Fig. 2). The study participants had a sex ratio of 0.7 (male: *n* = 293, female: *n* = 384), and a median age of 16 years (range 1 to 81 years). Among the 677 participants, 26 individuals were tested positive for HBsAg, leading to an estimated HBsAg prevalence of 3.8% [95% CI 2.6–5.7]. Children aged 5 years and younger showed the highest prevalence at 10.1% ([95% CI 4.9–18.2], *n* = 8/79; [OR = 3.8, 95% CI 1.4–10.6], *p* = 0.019) compared to adults aged 18 years and older (2.8% [95% CI 1.4–5.3], *n* = 8/282). There was no significant difference in HBsAg positivity between males (3.8% [95% CI 2.0–6.4]; *n* = 11/293) and females (3.9% [95% CI 2.3–6.2]; *n* = 15/384) (Table 1).

**Table 1:** HBsAg positivity rates in the study population

A. Positivity rates by survey tier						
Group	Total	HBsAg positivity			OR [CI 95%]	*p*-value
		*n*	%	CI 95%		
First-tier survey	74	17	23.0	14.5–33.5	Ref	–
Second-tier survey	677	26	3.8	2.6–5.7	5.8 [2.9–11.1]	< 0.001

B. Positivity rates among first-tier survey participants						
Characteristics	Total	HBsAg positivity			OR [CI 95%]	*p*-value
		*n*	%	CI 95%		
Age group (in years)						
≤ 5	79	8	10.1	4.9–18.2	3.8 [1.4–10.6]	0.019
6 to 17	316	10	3.2	1.6–5.5	1.1 [0.4–2.8]	0.999
≥ 18	282	8	2.8	1.4–5.3	Ref	–
Gender						
Male	293	11	3.8	2.0–6.4	1.0 [0.4–2.3]	0.926
Female	384	15	3.9	2.3–6.2	Ref	–

First-tier: index cases screening

Second-tier: household contacts screening

*HBsAg* Hepatitis B surface antigen

### Intra-familial survey (among familial contacts of index cases)

Of the 26 index households, five had relocated and six declined to participate, leaving 15 households in which HBsAg screening was ultimately conducted (Fig. 2). From each of these 15 households, five case contacts were included, except for family #24, where only four individuals participated. In total, 74 individuals (11 fathers, 12 mothers, and 51 children) were included in the intra-familial screening, of whom 17 tested positive for HBsAg, resulting in a prevalence of 23.0% (95% CI 14.5–33.5). Compared to the prevalence observed in the community survey, the prevalence among familial contacts of index cases was significantly higher (23.0% vs 3.8%, OR = 5.8 [95% CI 2.9–11.1]; *p* < 0.001) (Table 1).

Among the participants, 1.1% (*n* = 8/751) reported having been tested for hepatitis B in the past, and all recalled receiving negative results without having taken antiviral treatment. Only 10% of participants had heard of hepatitis B, typically referring to it using the local terms *mutshima muhula* (hepatomegaly) or *difu diuhula* (abdominal bloating). The majority of participants who tested positive were asymptomatic at the time of the surveys. However, 23.2% (*n* = 10/43) presented with at least one clinical sign consistent with viral hepatitis, including unexplained fatigue (*n* = 7; 16.3%), unexplained weight loss (*n* = 8; 18.6%), mucocutaneous jaundice (*n* = 5; 11.6%), abdominal bloating (*n* = 3; 7.0%), hepatomegaly (*n* = 3; 7.0%), and lower limb edema (*n* = 2; 4.6%).

### Molecular profile of HBV strains circulating in the study population

**Fig. 3:**
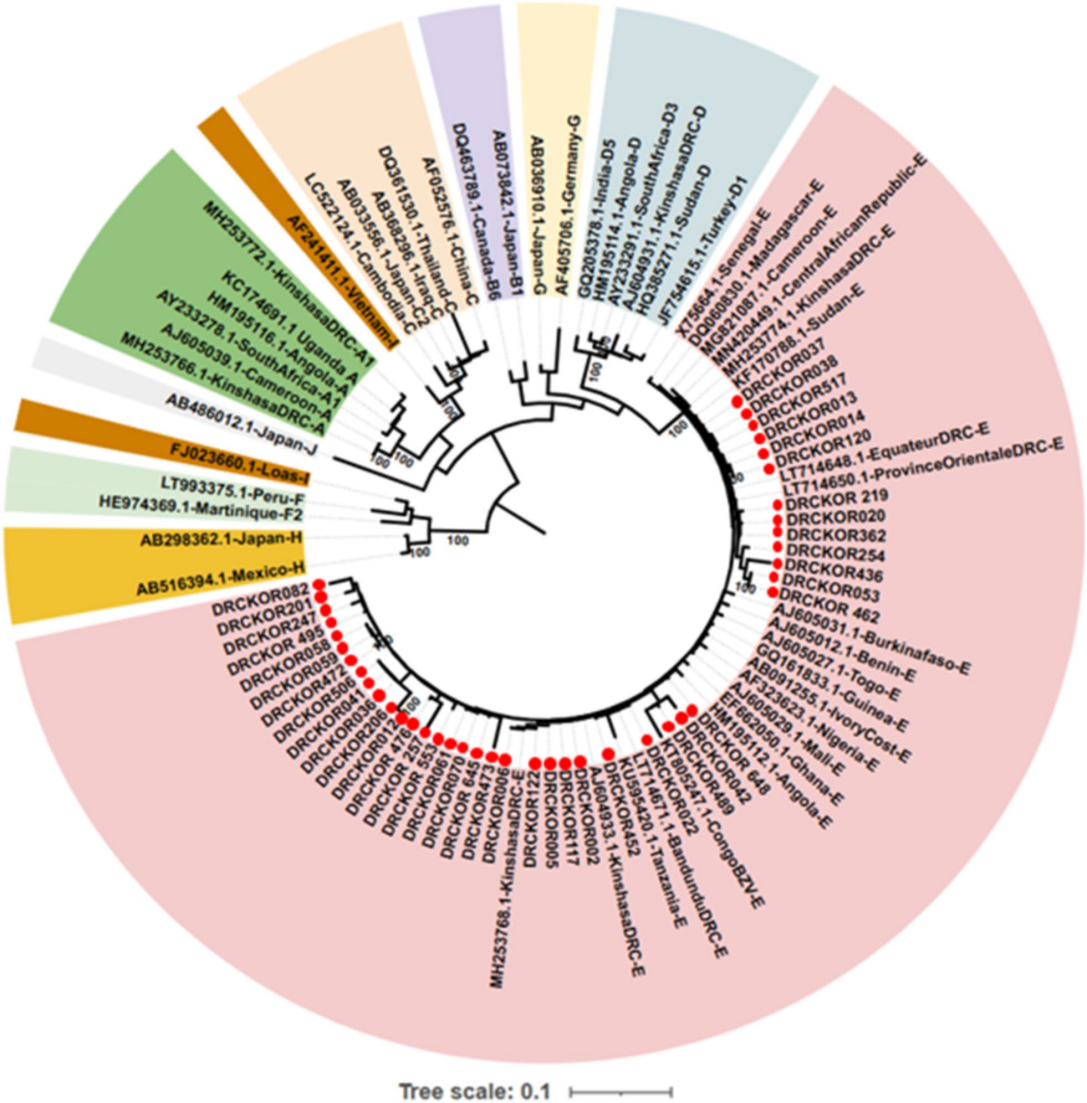
Maximum likelihood phylogenetic tree of HBV strains circulating in the study population. This rooted maximum likelihood phylogenetic tree was created using 92 complete preS/S sequences. These include 50 reference sequences from GenBank, representing all HBV genotypes (A–J), with accession numbers, country of origin, and genotype labels provided. Additionally, 42 sequences from this study are marked with red dots. The branch lengths are proportional to sequence divergence. Sequences of the same genotype are color-coded, with the red clade highlighting the predominant genotype E. The optimal substitution model, identified by IQ-TREE, is the transversion model with equal base frequencies and a discrete gamma distribution with four rate categories (TVMe + G4). Bootstrap values are shown at the tree nodes

A total of 42 HBV preS/S sequences were successfully obtained, including 25 from the 26 index cases and all 17 HBsAg-positive family contacts. All isolates were assigned to HBV genotype E (HBV/E) (Fig. 3). Notably, 41 of 42 HBV/E strains (97.6%) were classified as serotype *ayw4*, demonstrating high serotype concordance. One strain exhibited a discordant, yet-unclassified serotype, with an L127Q substitution alongside wild-type R122 and K160 positions. A detailed mutational analysis is provided in Supplementary Table S2. Furthermore, mutational analysis identified six mutations (i.e., P120S, C121Y, G130R, G145R, C147S, and T126N) within the “a” determinant of the major hydrophilic region of the HBsAg in 4 of 42 viral strains (9.5%), which have been reported as potentially associated with immune escape. Additionally, two mutations, rtI169T and rtM204K, were each detected in a different sequence (4.7%), within the overlapping RT gene, with previous reports suggesting possible links to antiviral drug resistance [8, 9]. Whole-genome amplification was successful in 96.1% (25/26) of index case samples, and phylogenetic reconstruction of whole-genomic sequences revealed close ancestral relationships, consistent with that of the preS/S region (Supplementary Figure S1).

### HBV transmission dynamics within studied families and community

**Fig. 4:**
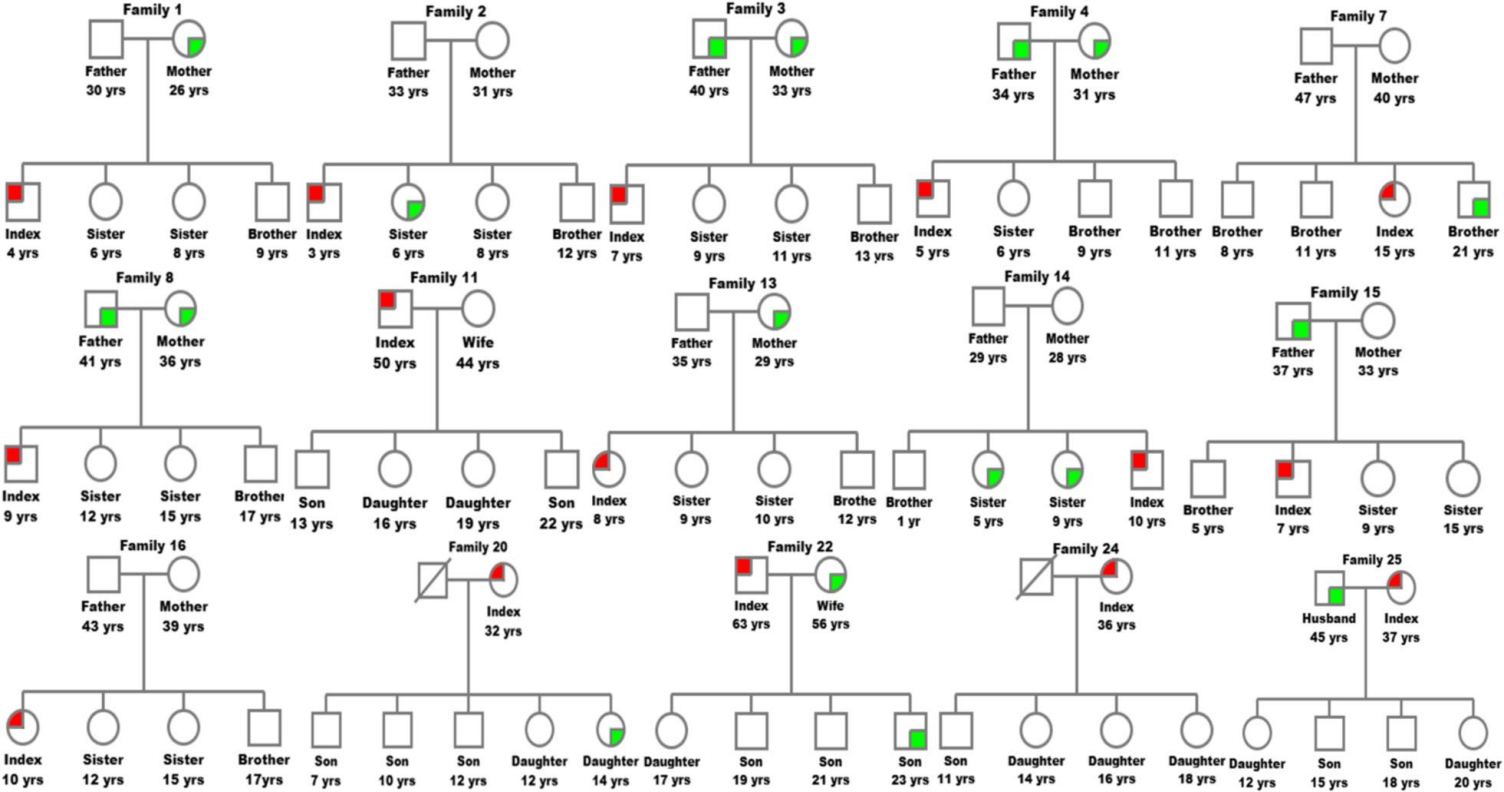
Family pedigree reconstruction for households included in the study. Squares represent males, and circles represent females. The first level of the tree shows the parents, while the second level includes their direct descendants. Red symbols indicate index cases positive for HBV, and green symbols represent positive family contacts. A diagonal line through a square indicates a deceased father. “yrs” refers to years

**Fig. 5:**
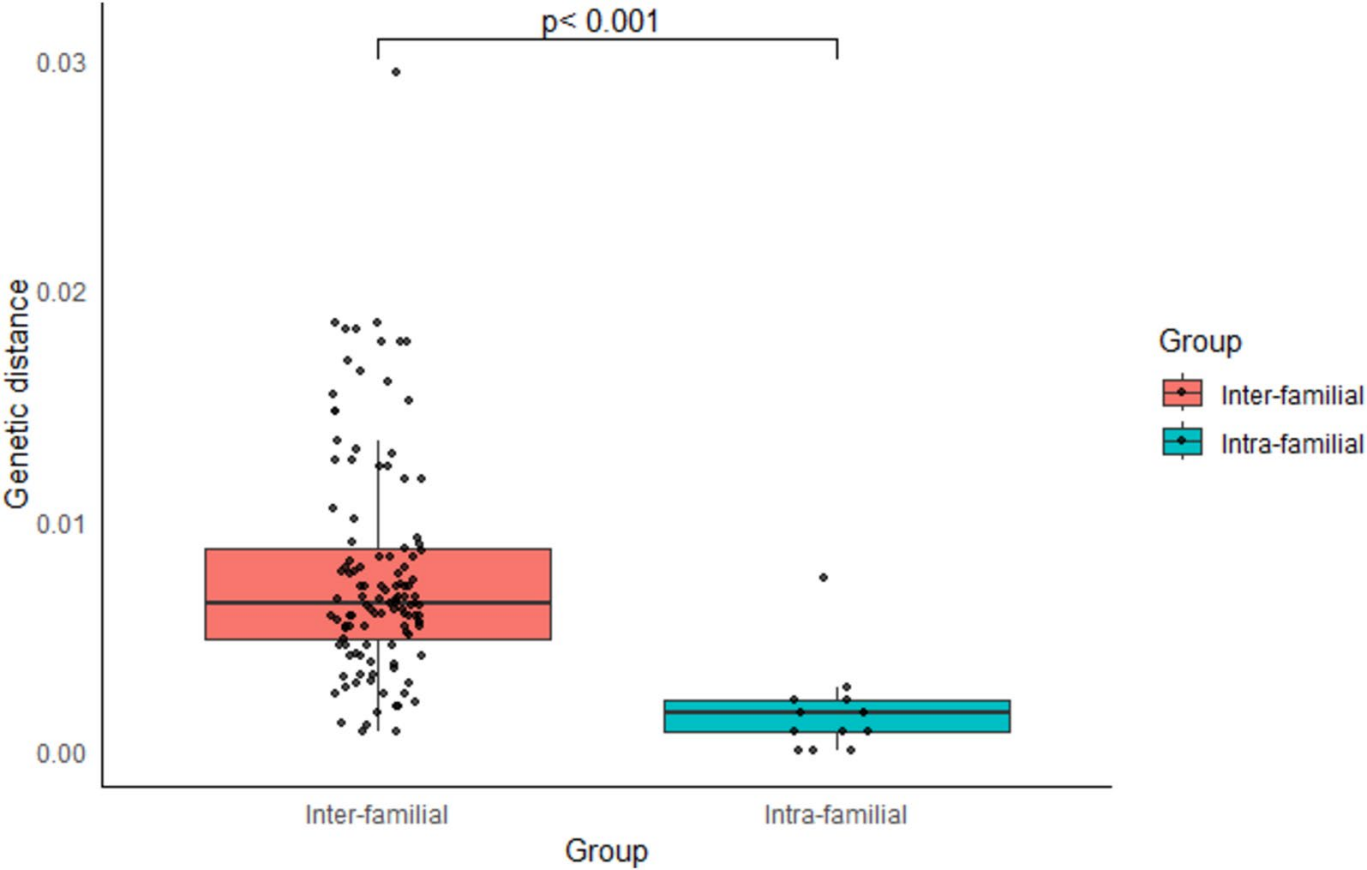
Mean inter- and intra-familial genetic distances. Boxplots of genetic distances: (orange) inter-familial distances and (cyan) intra-familial distances. The Wilcoxon rank-sum test was used to compare genetic distances of HBV within and between families, with a *p*-value < 0.001. Black individual plots indicate genetic distance specific to each family

**Fig. 6:**
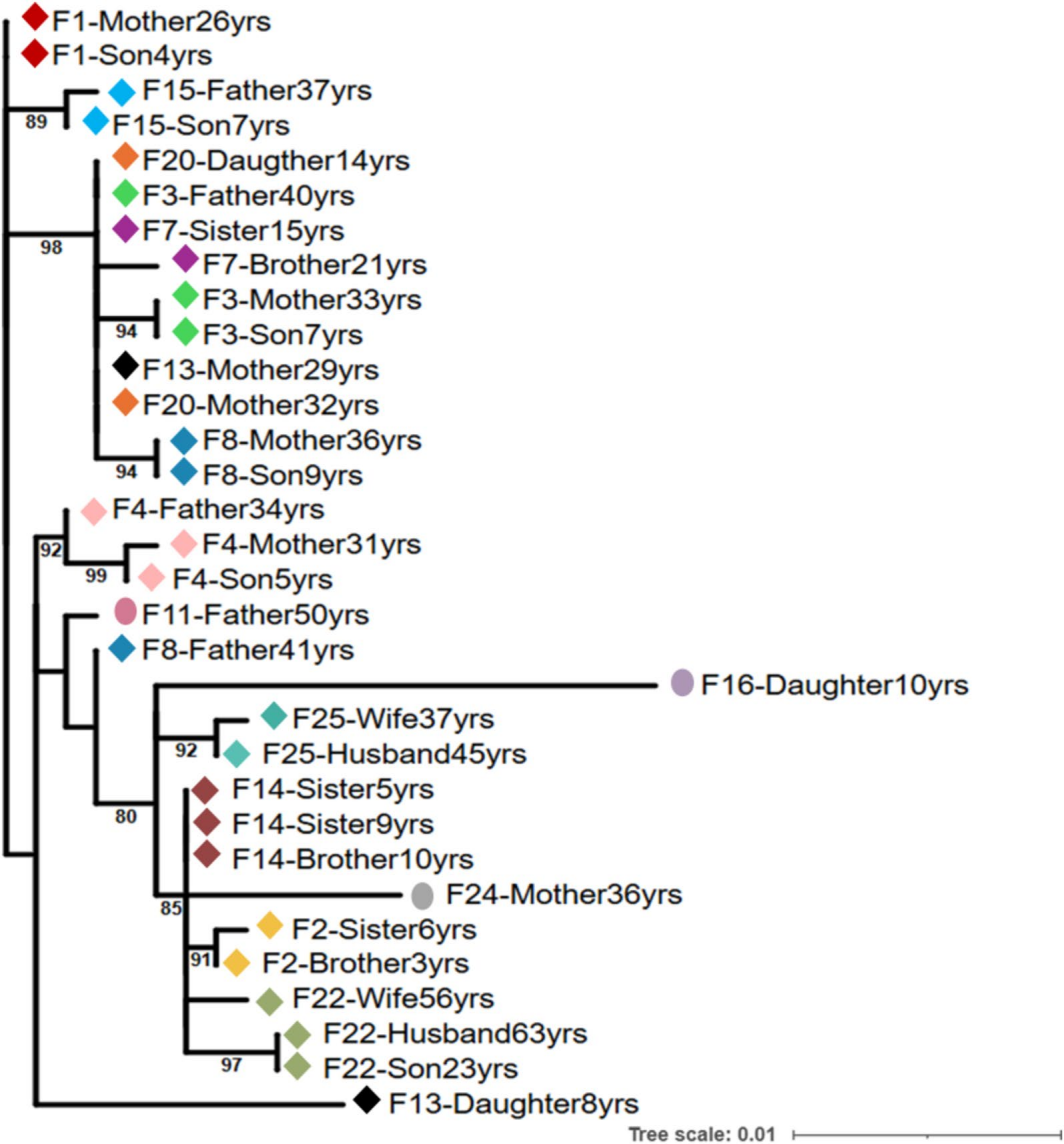
Maximum likelihood phylogenetic tree of preS/S sequences from local HBV isolates. This unrooted phylogenetic tree was constructed using the maximum likelihood method, based on 32 complete preS/S sequences from this study. The best-fit model, identified by IQ-TREE, is the Jukes–Cantor substitution model with invariant sites (JC + I). Families with more than two infected individuals are represented by colored diamonds, while families with only one infected individual are shown as circles. Sample labels include “F” for family, followed by the family number, relationship, and age. Each diamond and circle is uniquely colored to represent a different family. Bootstrap values are shown at the tree nodes

To explore transmission patterns, phylogenetic analysis, genetic distance calculations, and family pedigree reconstructions were employed using preS/S sequences. Among the 15 HBV-positive families studied, 73.3% (11/15) of families had an infected parent; maternal and paternal infections were present in 60% (9/15) and 53.8% (7/13) of the families, respectively, and 38.4% (5/13) had both parents infected (Fig. 4). Within families, genetic distances were significantly smaller in preS/S sequences than those between unrelated families (0.1 [range: 0–0.7] vs. 0.8 [range: 0.1–2.9]; *p* < 0.001), as shown in Fig. 5. Multiple infections were detected in 80% (12/15) of the households. In 66.7% (10/15) of these families, sequences clustered monophyletically (≥ established best cutoff value of 99.65% similarity) (Fig. 6). These findings suggest a predominant mode of HBV intra-familial transmission, including mother–child (26.7% [*n* = 4/15]; families #1, #3, #8 and #20), father–child (15.4% [*n* = 2/13]; families #15 and #22), sibling-to-sibling (20% [*n* = 3/15]; families #2, #7, and #14), and spouse-to-spouse (7.6% [*n* = 1/13]; family #25) patterns.

In contrast, 26.7% (4/15) of families harbored multiple infected individuals carrying genetically divergent HBV strains with sequence similarity < 99.65%, suggesting independent viral introductions rather than intra-familial transmission. In families #3 and #8, the parents were infected with distinct strains, but the mother–child pairs shared closely related strains, indicating possible mother-to-child transmission. In family #22, the parents carried divergent strains, but the father and child shared closely related strains, suggesting a possible father-to-child transmission. In family #13, the mother and child were infected with genetically distant strains, suggesting acquisition from separate sources. Additionally, HBV strains from families #3, #7, #13, and #20 formed a distinct monophyletic cluster (Fig. 6).

## Discussion

This study assessed the epidemiological landscape and transmission dynamics of HBV in Lukelenge, a peri-urban community of central DRC. Two key findings emerged: first, the region exhibited intermediate HBV endemicity, characterized by a markedly high prevalence among children aged 5 years and younger. Second, phylogenetic analysis combined with family pedigree reconstructions strongly suggested that intra-familial spread is the predominant mode of HBV transmission, particularly involving parent-to-child and sibling-to-sibling routes. These findings offer cornerstone evidence to guide local public health strategies.

Overall, the estimated HBsAg prevalence was 3.8% (95% CI 2.6–5.5; *n* = 26/677) in the study population, slightly higher than the national estimate of 3.3% for the DRC [6]. This positions the region within the WHO’s intermediate endemicity category for HBV, indicating a persistent infection burden [33]. Limited access to healthcare services, insufficient screening programs, and delays in diagnosis likely contribute to this situation [3–5, 16]. The high rate of infections observed among young children was of particular concern. One in ten children under the age of five tested positive for HBV, suggesting substantial early-life transmission. This is especially alarming given the well-established association between early-age infection and the risk of developing chronic HBV infection, cirrhosis, and HCC later in life [34]. Similar trends of elevated childhood HBV prevalence have also been reported in other sub-Saharan African countries, including Cameroon, South Africa, and Nigeria [16, 35, 36].

These findings likely result from significant evidence-practice gaps in HBV prevention efforts. Despite the availability of effective vaccines, the national HepB3 coverage in the DRC remains suboptimal, estimated at just 65% by the WHO in 2024 [37]. Moreover, the 2023–2024 DRC’s Demographic and Health Survey (DHS) reported that the coverage of the pentavalent DTP-HepB3-Hib vaccine is only 51.7% in Kasai-Oriental, where this study was conducted [38]. More critically, the country has yet to implement a universal, timely birth-dose HBV vaccination program [37], an essential measure for preventing mother-to-child transmission.

Limited vaccination coverage may have been further exacerbated by a broader decline in routine immunization, affecting all basic childhood vaccines in the country. According to the 2023–2024 DHS, basic vaccination coverage among children aged 12 to 23 months fell sharply from 45 to 21% [38]. In Kasai-Oriental, this fall reached 26% of children who were fully vaccinated by the time this study was conducted [38]. This decline has been largely attributed to disruptions of immunization activities caused by the COVID-19 pandemic, which significantly reduced vaccine delivery and demand across more than 100 countries, including the DRC [39]. These interruptions may partly explain the marked difference in HBV infection rates between under-five children and those aged 6–17 years in this study. Additionally, structural and socioeconomic barriers such as out-of-pocket costs for accessing vaccines or associated services have recently become a major obstacle for many families [40].

To reduce the HBV burden and prevent new infections, urgent public health interventions are needed. These should include the introduction of a universal birth-dose HBV vaccine program, the integration of systematic HBV screening during antenatal care, and access to antiviral prophylaxis for HBV-infected pregnant women. Strengthening health financing mechanisms and improving equitable access to vaccines across regions will also be critical for reversing declining coverage rates and protecting future generations from HBV-related disease.

Regarding HBV genetic characterization, this study exclusively identified the HBV genotype E and the *ayw4* serotype in the study population. This genotype has been previously described in the southeastern region of the DRC [19] and is known to be prevalent across Central and West Africa, including countries such as Cameroon and The Gambia [16, 41, 42]. The observed restricted genetic diversity suggests localized transmission patterns. Interestingly, the unique L127Q substitution found in one isolate suggests a potential emergence of yet-unclassified serotype. Located in the S gene, this mutation could affect antigenicity, immune recognition, and diagnostic accuracy, and warrants further investigation. Immune and vaccine escape mutations were identified in approximately one out of ten viral strains, including G145R, P120S, and T126N, which have been reported to be associated with reduced vaccine efficacy [43, 44]. The G145R mutation, in particular, is notable for its stability and ability to be transmitted both vertically and horizontally [15, 44]. These mutations enable HBV to evade immune responses from vaccination, immunoglobulin treatments, and natural immunity, leading to breakthrough infections and diminished vaccine effectiveness [15]. The detection of vaccine escape variants in this endemic area raises concerns about the risk of breakthrough infections, as seen previously in the DRC among vaccinated children aged 6 to 59 months [45]. Additionally, mutations such as rtI169T and rtM204K, possibly linked to antiviral resistance, were detected in nearly 5% of viral strains. An isolated rtI169T mutation may contribute to entecavir resistance when coexisting with additional mutations [46]. The rtM204K mutation confers resistance to adefovir and tenofovir in vitro, although this has not been confirmed in clinical outcomes [47]. This resistance could limit treatment options for chronic HBV infection, particularly in resource-limited settings where these drugs are commonly used [48]. The presence of drug-resistant strains in an antiviral treatment-naïve population presents a significant challenge for HBV control [49]. Regardless of the presence of these mutations, there is an urgent need for comprehensive control strategies, including enhanced viral surveillance, expanded access to antiviral treatments, and post-exposure prophylaxis.

This study provided also compelling evidence pointing towards the household as the primary setting for HBV transmission in the study area. Approximately 80% of affected families had multiple infected members, and genetically similar strains were detected within 66.7% of households, supporting the predominance of intra-familial transmission of HBV in the study community. Parent-to-child transmission emerged as the most common route, with maternal transmission likely playing a more significant role than paternal transmission. This aligns with findings from other HBV-endemic regions where perinatal and early childhood exposure are recognized as major contributors to the persistence of HBV infection [50]. Sibling-to-sibling transmission was also evident, likely facilitated by close contact and shared personal items, which are well-documented transmission routes in endemic settings [51]. Although spousal transmission was identified in one family, most infections appeared to be acquired during childhood, suggesting a limited local role for sexual transmission in the study community. Remarkably, horizontal acquisition of HBV through extra-familial route was limited, suggesting that exposure within the broader community plays a less significant role in its spread.

“We also observed that HBV strains from families #3, #7, #13, and #20 formed a distinct monophyletic cluster. In the Lukelenge HD, families often live within the same compound or in nearby houses, but even when they reside a block or two apart, strong social ties are maintained. These ties include the regular sharing of household items, frequent visits, and communal activities. Such close interactions likely promote intra-community transmission of HBV, which may explain the phylogenetic clustering observed among these families.”

Collectively, these findings provide important public health implications, indicating that targeted interventions within households—such as routine HBV screening for family members of infected individuals and the identification of HBsAg-positive individuals who may be eligible for antiviral therapy—could effectively reduce HBV disease burden [52]. Particularly given the growing momentum to integrate antenatal HBsAg screening into the triple elimination initiative for HIV, syphilis, and hepatitis B, this approach presents a valuable opportunity. Antenatal maternal screening could serve as an effective entry point for identifying infected households, enabling timely familial screening and follow-up interventions, as has been suggested in similar settings such as Burkina Faso [53].

This study has the following limitations. First, being a community-based survey conducted in a single location, the findings may not be generalizable to other regions. Second, the limited number of tested family contacts may have led to over- or under-estimations of intra-familial HBV prevalence. Third, the individual vaccination status of children was not assessed due to issues with parental recall, missing records, and limited knowledge about HBV. However, despite the existing national immunization program, the vaccination coverage was assumed to remain low. HIV status was not determined. Fourth, the cross-sectional design of the study precludes the determination of infection chronology within families, making it difficult to identify HBV transmission directions and primary sources. Fifth, genetic analyses were limited to the preS/S region of the HBsAg; this could limit insights into broader HBV genetic diversity and evolutionary patterns. Lastly, reliance on self-reported data may have introduced recall bias.

## Conclusions

This study provides valuable insights into HBV epidemiology in the central DRC with significant public health implications. The findings confirm an intermediate endemicity profile, with transmission occurring predominantly within families through vertical (parent-to-child) and horizontal (sibling-to-sibling) routes. The exclusive detection of HBV genotype E, *ayw4* serotype, and limited genetic diversity suggest stable, localized transmission patterns. The identification of immune escape and possible drug-resistant variants highlights the need for improved HBV surveillance, enhanced vaccination strategies, and expanded access to antiviral treatment. Moving forward, targeted interventions, including universal birth-dose vaccination, maternal screening, and household-based prevention measures, will be essential in reducing the HBV burden in the study population.

## Supplementary Information

Supplementary Materia 1.

## Data Availability

No datasets were generated or analyzed during the current study.

## Abbreviations

BIC: Bayesian Information Criterion
CI: Confidence intervals
DRC: Democratic Republic of Congo
HBsAg: Hepatitis B surface antigen
HBV/E: HBV genotype E
HBV: Hepatitis B virus
HCC: Hepatocellular carcinoma
HD: Health District
HepB3: Three-dose hepatitis B vaccination series
INRB: Institut National de Recherche Biomédicale
iTOL: Interactive Tree of Life
K2P: Kimura-2-Parameter
OR: Odd Ratio
ORFs: Open reading frames
PCR: Polymerase chain reaction
ROC: Receiver operating characteristic
WHO: World Health Organization

## References

[1] Easterbrook PJ, Luhmann N, Bajis S, Min MS, Newman M, Lesi O, et al. WHO 2024 hepatitis B guidelines: an opportunity to transform care. Lancet Gastroenterol Hepatol. 2024;9(6):493–5. 38614110 10.1016/S2468-1253(24)00089-X

[2] World Health Organization. Global health sector strategy on viral hepatitis 2016–2021: towards ending viral hepatitis. Geneva: WHO; 2016.

[3] World Health Organization. Global hepatitis report 2024: action for access in low- and middle-income countries. Geneva: WHO; 2024.

[4] Sonderup MW, Spearman CW. HBV elimination in Africa—Current status and challenges. Clin Liver Dis. 2024;23(1): e0166. 10.1097/CLD.0000000000000166 PMC11068139 38707243

[5] Sonderup MW, Spearman CW. Global disparities in hepatitis B elimination—a focus on Africa. Viruses. 2022;14(1):82. 35062286 10.3390/v14010082 PMC8777803

[6] Thompson P, Parr JB, Holzmayer V, Carrel M, Tshefu A, Mwandagalirwa K, et al. Seroepidemiology of hepatitis B in the Democratic Republic of the Congo. Am J Trop Med Hyg. 2019;101(1):226. 31074406 10.4269/ajtmh.18-0883 PMC6609197

[7] Thompson P, Morgan CE, Ngimbi P, Mwandagalirwa K, Ravelomanana NLR, Tabala M, et al. Arresting vertical transmission of hepatitis B virus (AVERT-HBV) in pregnant women and their neonates in the Democratic Republic of the Congo: a feasibility study. Lancet Glob Health. 2021;9(11):e1600–9. 34416175 10.1016/S2214-109X(21)00304-1 PMC8607275

[8] Kabore HJ, Li X, Alleman MM, Manzengo CM, Mumba M, Biey J, et al. Morbidity and mortality weekly report progress toward hepatitis b control and elimination of mother-to-child transmission of hepatitis B virus-World Health Organization African Region, 2016–2021. Centers Dis Control Prev. 2023;72(29):2016–21. 10.15585/mmwr.mm7229a2 PMC10360654 37471264

[9] Anderson M, Mangogola T, Phinius BB, Mpebe G, Aimakhu CO, Choga WT, et al. Hepatitis B virus prevalence among HIV-uninfected people living in rural and peri-urban areas in Botswana. Microorganisms. 2024;12(6): 1207. 38930589 10.3390/microorganisms12061207 PMC11205512

[10] Nlombi CM, Natuhoyila AN, Tudiakuilayi MM. Evaluation of seroprevalence and factors associated with the portage of HBsAg in pregnant women in rural Vanga and semi-urban Maluku. Int J Health Sci Res. 2019;9(12):1–9.

[11] Moutchia J, Njouom R, Rumpler E, Besombes C, Texier G, Tejiokem M, et al. Maternal age at first childbirth and geographical variation in HBV prevalence in Cameroon: important role of mother-to-child transmission. Clin Infect Dis. 2022;74:836–45. 34125878 10.1093/cid/ciab548

[12] Whittle H, Inskip H, Bradley AK, McLaughlan K, Shenton F, Lamb W, et al. The pattern of childhood hepatitis B infection in two Gambian villages. J Infect Dis. 1990;161:1112–5. 10.1093/infdis/161.6.1112. 2345294 10.1093/infdis/161.6.1112

[13] Ansari A, Vincent JP, Moorhouse L, Shimakawa Y, Nayagam S. Risk of early horizontal transmission of hepatitis B virus in children of uninfected mothers in sub-Saharan Africa: a systematic review and meta-analysis. Lancet Glob Health. 2023;11(5):e715–28. 37061310 10.1016/S2214-109X(23)00131-6

[14] Morgan CE, Ngimbi P, Boisson-Walsh AJN, Ntambua S, Matondo J, Tabala M, et al. Hepatitis B virus prevalence and transmission in the households of pregnant women in Kinshasa, Democratic Republic of Congo. Open Forum Infect Dis. 2024;11:1–10. 10.1093/ofid/ofae150 PMC11017325 38623568

[15] Pollicino T, Cacciola I, Saffioti F, Raimondo G. Hepatitis B virus PreS/S gene variants: pathobiology and clinical implications. J Hepatol. 2014;61(2):408–17. 10.1016/j.jhep.2014.04.041. 24801416 10.1016/j.jhep.2014.04.041

[16] Pinho-nascimento CA, Bratschi MW, Soares CC, Warryn L, Minyem JC, Terezinha M, et al. crossm transmission of hepatitis B and D viruses in an African rural. Am Soc Microbiol. 2018;3(5):1–15. 10.1128/mSystems.00120-18 PMC6143728 30246145

[17] Chen J, Li L, Yin Q, Shen T. A review of epidemiology and clinical relevance of Hepatitis B virus genotypes and subgenotypes. Clin Res Hepatol Gastroenterol. 2023. 10.1016/j.clinre.2023.102180. 37479136 10.1016/j.clinre.2023.102180

[18] Chen BF. Hepatitis B virus pre-S/S variants in liver diseases. World J Gastroenterol. 2018;24(14):1507–20. 29662289 10.3748/wjg.v24.i14.1507 PMC5897855

[19] Kabamba AT, Kalunga BT, Mwamba CM, Nyembo CM, Dufrasne F, Dessilly G, et al. Epidemiological aspects and molecular characterization of the hepatitis B virus among blood donors in Lubumbashi, Democratic Republic of Congo. Transfus Clin Biol. 2021;28:30–7. 33232802 10.1016/j.tracli.2020.10.012

[20] Shindano TA, Horsmans Y, Kabamba BM. Genetic and phylogenic characterization of hepatitis B virus in the eastern part of the Democratic Republic of Congo. J Med Virol. 2018;90:250–4. 28460156 10.1002/jmv.24837

[21] Ministère de la Santé Publique, République Démocratique du Congo. Enquête nutritionnelle anthropométrique et de mortalité selon la méthodologie SMART. 2023:1–9.

[22] Nanjundeswaraswamy TS, Divakar S. Determination of sample size and sampling methods in applied research. Proc Eng Sci. 2021;3:25–32.

[23] Zhang Q, Wu G, Richards E, Jia S, Zeng C. Universal primers for HBV genome DNA amplification across subtypes: a case study for designing more effective viral primers. Virol J. 2007;4:1–7. 17892576 10.1186/1743-422X-4-92 PMC2099425

[24] Technelysium Pty Ltd. Chromas. Brisbane (Australia): Technelysium Pty Ltd; https://technelysium.com.au/wp/chromas/

[25] Katoh K, Standley DM. MAFFT multiple sequence alignment software version 7: improvements in performance and usability. Mol Biol Evol. 2013;30:772–80. 23329690 10.1093/molbev/mst010 PMC3603318

[26] Minh BQ, Schmidt HA, Chernomor O, Schrempf D, Woodhams MD, Von Haeseler A, et al. IQ-tree 2: new models and efficient methods for phylogenetic inference in the genomic era. Mol Biol Evol. 2020;37:1530. 32011700 10.1093/molbev/msaa015 PMC7182206

[27] Letunic I, Bork P. Interactive Tree of Life (iTOL) v6: recent updates to the phylogenetic tree display and annotation tool. Nucleic Acids Res. 2024;52:W78-82. 10.1093/nar/gkae268. 38613393 10.1093/nar/gkae268 PMC11223838

[28] Genome Detective. Hepatitis B virus phylogenetic typing tool. https://www.genomedetective.com/app/typingtool/hbv/how-to-use

[29] Tamura K, Stecher G, Kumar S. MEGA11: molecular evolutionary genetics analysis version 11. Mol Biol Evol. 2021;38:3022–7. 10.1093/molbev/msab120. 33892491 10.1093/molbev/msab120 PMC8233496

[30] Bell TG, Kramvis A. Bioinformatics tools for small genomes, such as Hepatitis B virus. Viruses. 2015;7: 781. 25690798 10.3390/v7020781 PMC4353916

[31] HIV-GRADE - HIV-GRADE viral Genotypic resistance algorithms. https://www.hiv-grade.de/cms/grade/.

[32] The R Development Core Team. R: a language and environment for statistical computing. Vienna, Austria: R Foundation for Statistical Computing; 2024. https://www.R-project.org/.

[33] Schweitzer A, Horn J, Mikolajczyk RT, Krause G, Ott JJ. Estimations of worldwide prevalence of chronic hepatitis B virus infection: a systematic review of data published between 1965 and 2013. Lancet. 2015;386:1546–55. 26231459 10.1016/S0140-6736(15)61412-X

[34] Shimakawa Y, Yan HJ, Tsuchiya N, Bottomley C, Hall AJ. Association of early age at establishment of chronic Hepatitis B infection with persistent viral replication, liver cirrhosis and hepatocellular carcinoma: a systematic review. PLoS ONE. 2013;8: e69430. 23894479 10.1371/journal.pone.0069430 PMC3716646

[35] Moonsamy S, Suchard M, Pillay P, Prabdial-Sing N. Prevalence and incidence rates of laboratory-confirmed hepatitis B infection in South Africa, 2015 to 2019. BMC Public Health. 2022;22:1–13. 10.1186/s12889-021-12391-3. 34991533 10.1186/s12889-021-12391-3 PMC8739689

[36] Ezeilo MC, Engwa GA, Iroha RI, Odimegwu DC. Seroprevalence and associated risk factors of hepatitis B virus infection among children in Enugu metropolis. Virol Res Treat. 2018;9: 0–6. 10.1177/1178122X18792859 PMC6108015 30150873

[37] World Health Organization. Hepatitis B vaccination coverage: WHO/UNICEF Estimates of National Immunization Coverage (WUENIC). Geneva: WHO; 2024.

[38] République Démocratique du Congo. Enquête Démographique et de Santé (EDS-RDC III) 2023–2024: rapport final. Kinshasa (RDC): Institut National de la Statistique, Ecole de Santé Publique de Kinshasa; 2025. 955 p.

[39] Organisation mondiale de la Santé. Soutenir « le grand rattrapage » des enfants zéro dose et sous-vaccinés en République démocratique du Congo. 2023 août 10.

[40] Université de Kinshasa, Ecole de Santé Publique. Dépenses des ménages pour la vaccination des enfants en République Démocratique du Congo : analyse du lien avec la couverture vaccinale des enfants de 6 à 23 mois. Note d’information. Enquête de couverture vaccinale ECV-RDC 2023/04. Kinshasa (RDC) : Université de Kinshasa; 2023.

[41] Kafeero HM, Ndagire D, Ocama P, Kato CD, Wampande E, Walusansa A, et al. Mapping hepatitis B virus genotypes on the African continent from 1997 to 2021: a systematic review with meta-analysis. Sci Rep. 2023;13:1–14. 10.1038/s41598-023-32865-1. 37029173 10.1038/s41598-023-32865-1 PMC10082212

[42] Dumpis U, Holmes EC, Mendy M, Hill A, Thursz M, Hall A, et al. Transmission of hepatitis B virus infection in Gambian families revealed by phylogenetic analysis. J Hepatol. 2001;35:99–104. 11495049 10.1016/s0168-8278(01)00064-2

[43] Lazarevic I. Clinical implications of hepatitis B virus mutations: recent advances. World J Gastroenterol. 2014;20:7653–64. 24976703 10.3748/wjg.v20.i24.7653 PMC4069294

[44] Wang J, Zhu B, Lu M, Yang D. Hepatitis B virus preS/S gene mutations and their clinical implications. Ann Blood. 2017;2:17–17.

[45] Morgan CE, Powers KA, Edwards JK, et al. Characterizing hepatitis B virus infection in children in the Democratic Republic of Congo to inform elimination efforts. medRxiv. 2024. 10.1101/2024.06.12.24308840.39417127

[46] Caligiuri P, Cerruti R, Icardi G, Bruzzone B. Overview of hepatitis B virus mutations and their implications in the management of infection. World J Gastroenterol. 2016;22:145–54. 26755866 10.3748/wjg.v22.i1.145 PMC4698481

[47] Zhang K, Bach C, Fraune MN, Xia Y, Beggel B, Kaiser R, et al. Novel rtM204 mutations in HBV polymerase confer reduced susceptibility to adefovir and tenofovir. J Antivirals Antiretrovirals. 2017;09:10–7.

[48] Desalegn H, Aberra H, Berhe N, Mekasha B, Stene-Johansen K, Krarup H, et al. Treatment of chronic hepatitis B in sub-Saharan Africa: 1-year results of a pilot program in Ethiopia. BMC Med. 2018;16:1–10. 10.1186/s12916-018-1229-x PMC6296040 30554571

[49] Lumley SF, Mokaya J, Maponga TG, Kramvis A, Dusheiko G, Irving W, et al. Hepatitis B virus resistance to nucleos(t)ide analogue therapy: WHO consultation on questions, challenges, and a roadmap for the field. Lancet Microbe. 2025. 10.1016/j.lanmic.2025.101076. 40220768 10.1016/j.lanmic.2025.101076

[50] Pan CQ, Zhang JX. Natural history and clinical consequences of hepatitis B virus infection. Int J Med Sci. 2005;2:36–40. 15968338 10.7150/ijms.2.36 PMC1142223

[51] Martinson FEA, Weigle KA, Royce RA, Weber DJ, Suchindran CM, Lemon SM. Risk factors for horizontal transmission of hepatitis B virus in a rural district in Ghana. Am J Epidemiol. 1998;147:478–87. 9525535 10.1093/oxfordjournals.aje.a009474

[52] Zhao W. Guidelines for the prevention, diagnosis, care and treatment for people with chronic hepatitis B infection (text extract): executive summary. Infect Dis Immunity. 2024;4(3):103–5. 39391287 10.1097/ID9.0000000000000128 PMC11462912

[53] Guingané AN, Kaboré R, Shimakawa Y, Somé EN, Kania D, Pisoni A, et al. Screening for hepatitis B in partners and children of women positive for surface antigen, Burkina Faso. Bull World Health Organ. 2022;100:256–67. 35386558 10.2471/BLT.21.287015 PMC8958837

