# Supplementary material for: Intra-familial transmission of Hepatitis B virus in a peri-urban community from the Democratic Republic of the Congo
Source: Trop Med Health. 2025 Jul 28;53:99. doi: 10.1186/s41182-025-00781-x (PMC12302796; doi:10.1186/s41182-025-00781-x)
Supplement: Supplementary file 1 — Supplementary Materia 1. [file 41182_2025_781_MOESM1_ESM.docx]

**Intra-familial transmission of Hepatitis B virus in a peri-urban community from the Democratic Republic of the Congo**

Florence Cindibu Kalonji,MD^1,2^, Yu Nakagama,PhD^1^, Evariste Tshibangu-Kabamba,PhD^1,2^, Nadine Kayiba Kalenda, PhD^1,3^, Shun Nakagama,PhD^1^, Sachie Nakagama,MSc^1^, Pathy Kamanga Nkolongo,MD^2^, Nestor Kalala-Tshituka,MD^1,3^, Alphonse Lufuluabu Mpemba,MD^1,2^, Faustin Ndjibu Mpoji,MD^3^, André Kabongu Kalala,MD^3,^ Benjamin Muamba Mpoyi,MD^4^, Dieudonné Mumba Ngoyi,PhD^5^, Natsuko Kaku,PhD^1^, Yusuke Shimakawa,PhD^6^, Ghislain Tumba Disashi,PhD^2^, and Yasutoshi Kido,PhD^1^

^1^Department of Virology and Parasitology, Graduate School of Medicine, Osaka Metropolitan University, Osaka, Japan.

^2^Department of Internal Medicine, Faculty of Medicine – Pharmacy and Public Health, University of Mbujimayi, Mbujimayi, Democratic Republic of Congo.

^3^Department of Public Health, Faculty of Medicine – Pharmacy and Public Health, University of Mbujimayi, Mbujimayi, Democratic Republic of Congo.

^4^Secondary Hospital of Katanda, Kasai-Oriental, Democratic Republic of Congo

^5^Department of Parasitology, National Institute of Biomedical Research, Kinshasa, Democratic Republic of Congo

^6^ Institut Pasteur, Université Paris Cité, Unité d'Épidémiologie des Maladies Émergentes, Paris, France

**Corresponding Author**

Professor Yasutoshi Kido

E-mail: [kidoyasu@omu.ac.jp](mailto:kidoyasu@omu.ac.jp);

Tel.: +81-(0)6-6645-3761; FAX +81-(0)6-6645-3762

Institution: Department of Parasitology and Virology, Graduate School of Medicine, Osaka Metropolitan University

Address: 1-4-3 Asahi-cho, Abeno-ku, Osaka-shi, Osaka 545-8585 – Japan

**SUPPLEMENTARY MATERIAL**

**SUPPLEMENTARY TABLES**

**Table S1. Primers used for HBV genomic amplification and nucleotide sequencing**

| **Target region** | **Primers ID** | **5’-Sequence-3’** | **Direction** |
| --- | --- | --- | --- |
| HBV Whole genome | **For amplification** |  |  |
|  | WA-L [1] | ACTGTTCAAGCCTCCAAGCTGTGC | Forward |
|  | WA-R [1] | AGCAAAAAGTTGCATGGTGCTGGT | Reverse |
|  | **For sequencing** |  |  |
|  | WA-L [1] | ACTGTTCAAGCCTCCAAGCTGTGC | Forward |
|  | PS1 [2] | CCATATTCTTGGGAACAAGA |  |
|  | PS1* | CCTTATTCTTGGGAACACGA |  |
|  | S18 [2] | GGATGATGTGGTATTGGGGGCCA |  |
|  | S18* | GGATGATGTGGTATTGGGGGCGA |  |
|  | X1 [2] | ACCTCCTTTCCATGGCTGCT |  |
|  | WA-R [1] | AGCAAAAAGTTGCATGGTGCTGGT | Reverse |
|  | P3 [2] | AAAGCCCAAAAGACCCACAA |  |
|  | PS8 [2] | TTCCTGAACTGGAGCCACCA |  |
|  | PS8* | TTCCGGAACTGGAGGCCACCA |  |
|  | C8 [2] | GAGGGAGTTCTTCTTCTAGG |  |
|  | WA-R [1] | AGCAAAAAGTTGCATGGTGCTGGT |  |
| HBV preS/S | **For amplification** |  |  |
|  | PS1 [2] | CCATATTCTTGGGAACAAGA | Forward |
|  | P3 [2] | AAAGCCCAAAAGACCCACAA | Reverse |
|  | S2 [2] | GGGTTTAAATGTATACCCAAAGA | Forward |
|  | **For sequencing** |  |  |
|  | PS1 [2] | CCATATTCTTGGGAACAAGA | Forward |
|  | PS4 [2] | ACACTCATCCTCAGGCCATGCAGTG | Forward |
|  | S2 [2] | GGGTTTAAATGTATACCCAAAGA | Reverse |
| (*) Denotes modified nucleotides in primers. | | | |

**Table S2. HBsAg escape and possible antiviral resistance mutations in the 42 HBV/E preS/S sequences**

| **Participant ID** | **Age (yr)** | **Immune escape mutants** | **Possible drug-resistant mutants** |
| --- | --- | --- | --- |
| F21-Son | 1 | P120S and C121Y | - |
| F18-Son | 2 | G130R and G145R | - |
| F16-Daughter | 10 | C147S | - |
| F12-Mother | 24 | T126N and G145R | I169T |
| F13-Mother | 29 | - | M204K |
| Total |  | 4/42 (9.5%) | 2/42(4.7%) |

HBsAg: Hepatitis B surface antigen. ID: Identifier. ''F'': denotes family, followed by the family number. ''yr'': years.

**Table S3. Genetic variations in preS/S nucleotide sequences among family members**

| **Family ID** | **Position (nt)** | **Consensus** | **Infected family members** | | | **Divergence** |
| --- | --- | --- | --- | --- | --- | --- |
|  |  |  | **Member1** | **Member2** | **Member3** |  |
| F1 | - | - | Mother | Son | - | None |
| F14 | - | - | Sister1 | Sister2 | Brother | None |
| F20 | - | - | Mother | Daughter | - | None |
| F2 |  |  | Brother | Sister | - |  |
|  | 1187 | G | G | **T** | - |  |
| F15 |  |  | Son | Father | - | Yes |
|  | 385 | A | A | **G** | - |  |
| F25 |  |  | Wife | Husband | - | Yes |
|  | 495 | A | A | **G** | - |  |
| F7 |  |  | Sister | Brother | - |  |
|  | 1173 | A | A | **T** | - |  |
|  | 1188 | G | G | **T** | - |  |
| F4 |  |  | Son | Mother | Father | Yes |
|  | 371 | A | A | A | **C** |  |
|  | 374 | A | A | A | **C** |  |
|  | 412 | C | C | **A** | C |  |
| F3 |  |  | Son | Mother | Father | Yes |
|  | 404 | A | A | A | **T** |  |
|  | 1050 | A | A | A | **G** |  |
|  | 1126 | C | G | G | **C** |  |
| F22 |  |  | Husband | Wife | Son |  |
|  | 101 | G | G | **C** | G |  |
|  | 106 | A | A | **G** | A |  |
|  | 113 | G | G | **T** | G |  |
|  | 224 | A | A | **G** | A |  |
| F8 |  |  | Father | Mother | Son | Yes |
|  | 7 | C | C | **G** | **G** |  |
|  | 8 | T | T | **C** | **C** |  |
|  | 192 | G | G | **T** | **T** |  |
|  | 385 | G | **A** | G | G |  |
| F13 |  |  | Mother | Daughter | - | Yes |
|  | 957 | C | C | **T** | - |  |
|  | 958 | A | A | **T** | - |  |
|  | 959 | A | A | **G** | - |  |
|  | 1042 | A | A | **T** | - |  |
|  | 1050 | A | A | **T** | - |  |
|  | 1060 | A | A | **T** | - |  |
|  | 1106 | A | A | **T** | - |  |
|  | 1109 | A | A | **T** | - |  |
|  | 1159 | A | **C** | A | - |  |

**SUPPLEMENTARY FIGURES**

**
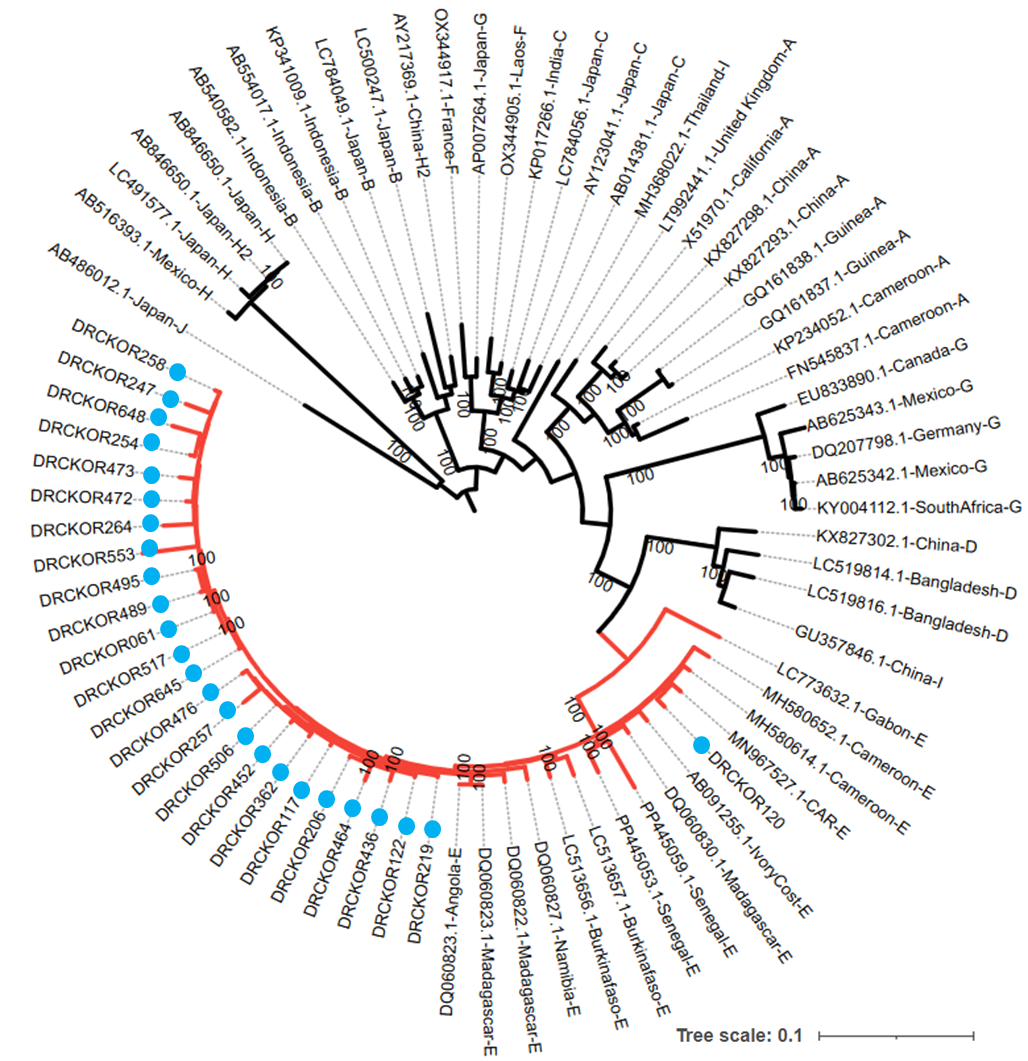
**

**Figure S1. Phylogenetic Analysis of the Complete HBV Genome Sequences**

A rooted maximum likelihood phylogenetic tree of 75 complete genome sequences, including 50 reference sequences covering all HBV genotypes (A-J) from the GenBank database (indicated by their accession numbers, country of origin, and genotype letter), as well as 25 sequences from this study indicated in blue dots. The branch lengths are proportional to the sequence divergence. The branches representing predominant genotype E sequences are shown in red. The best-fit substitution model, identified by IQ-TREE, was the general reversible mutations, empirical base frequencies, invariant sites, and rate variation across sites using three discrete categories (GTR+F+I+R3). Bootstrap values are shown at the nodes.


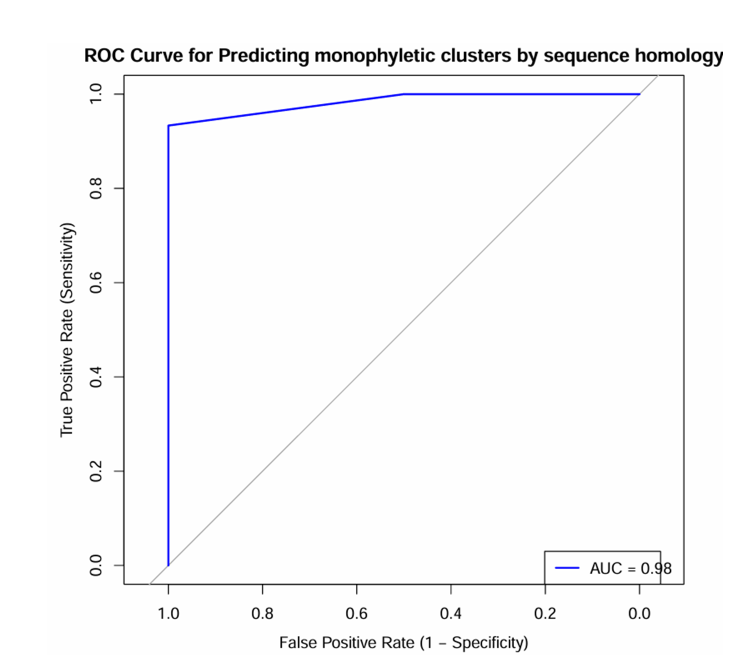


**Figure S2. Receiver Operating Characteristic (ROC) curve for predicting monophyletic clusters by sequence homology.** The curve demonstrates a high predictive performance with an Area Under the Curve (AUC) of 0.98, indicating strong discriminatory power.

**References**

1. Zhang Q, Wu G, Richards E, Jia S, Zeng C. Universal primers for HBV genome DNA amplification across subtypes: A case study for designing more effective viral primers. Virology Journal. 2007; 4:1–7.

2. Pinho-nascimento CA, Bratschi MW, Soares CC, et al. crossm Transmission of Hepatitis B and D Viruses in an African Rural. Amaerican Society For Microbiology. 2018; 3(5):1–15.
